# Identification of potential metabolic biomarkers of rectal cancer and of the effect of neoadjuvant radiochemotherapy

**DOI:** 10.1371/journal.pone.0250453

**Published:** 2021-04-22

**Authors:** Elisabet Rodríguez-Tomàs, Meritxell Arenas, Junior Gómez, Johana Acosta, Jordi Trilla, Yolanda López, Miguel Árquez, Laura Torres, Pablo Araguas, Anna Hernández-Aguilera, Gerard Baiges-Gaya, Helena Castañé, Jordi Camps, Jorge Joven

**Affiliations:** 1 Unitat de Recerca Biomèdica, Institut d’Investigació Sanitària Pere Virgili, Hospital Universitari de Sant Joan, Universitat Rovira i Virgili, Reus, Spain; 2 Department of Radiation Oncology, Institut d’Investigació Sanitària Pere Virgili, Hospital Universitari de Sant Joan, Universitat Rovira i Virgili, Reus, Spain; 3 Department of Pathology, Institut d’Investigació Sanitària Pere Virgili, Hospital Universitari de Sant Joan, Universitat Rovira i Virgili, Reus, Spain; Weill Cornell Medical College in Qatar, QATAR

## Abstract

We report a pilot study on the feasibility of determinations of circulating levels of paraoxonase-1 (PON1) and compounds related to energy metabolism as biomarkers for the evaluation of patients with rectal cancer (RC), and the effects produced by neoadjuvant radiochemotherapy (NRCT). We studied 32 patients treated with radiotherapy plus capecitabine concomitant chemotherapy and 48 control subjects. We identified pre-NRCT PON1 and α-ketoglutarate as the parameters that best discriminated between RC patients and the control group. Receiver operating characteristics analysis of the combination of the two parameters showed an area under the curve (AUC) of 0.918. Moreover, patients who presented a pathological complete response (pCR) to treatment had lower plasma pre-NRCT valine concentrations (AUC of 0.826). Patients who had a relapse had lower concentrations of succinate (AUC of 0.833). The results of the present study illustrate the usefulness of investigating alterations in oxidative stress and metabolism in RC. Due to the small number of patients studied, our results must be considered preliminary, but they suggest that the determination of circulating levels of PON1 and α-ketoglutarate might be a valuable tool for the early diagnosis of RC, while the determination of valine and succinate might effectively predict pCR and the appearance of relapse.

## Introduction

Rectal cancer (RC) is the eighth most common cancer worldwide. It is associated with high mortality and morbidity, and is becoming increasing prevalent in young individuals. Neoadjuvant radiochemotherapy (NRCT) is the standard treatment for patients with this disease [1–4]. However, data on the effects of this treatment on metabolism is scarce. It has been suggested that metabolic alterations are essential hallmarks in the development of carcinogenesis [5] and indeed, cancer metabolism has become an important subject of research in recent years. Examining the metabolic differences between cancer patients and healthy people can provide important information for the development of new therapeutic strategies and the discovery of new biomarkers for the diagnosis, prognosis and monitoring of the disease [6].

The development of targeted metabolomics has allowed researchers to use practical, precise methods for studying a large amount of metabolites in small samples of tissue, cell extracts, or biological fluids. Recent studies have underlined the important role of energy metabolism in tumour development and progression and have highlighted the key phenomenon that energy metabolism as a whole adapts to the high demands on protein, lipid and nucleic acid synthesis needed for tumour cell proliferation. Recent studies by our group reported that the plasma concentrations of products of glycolysis, citric acid cyclic and amino acid metabolism are considerably altered in patients with breast, lung, and head and neck cancer and that radiation therapy (RT) was a factor in rectifying these disturbances [7–9].

Metabolic alterations in cancer are related to oxidative stress [10]. One of the main systems that protects the cell from oxidative stress is paraoxonase-1 (PON1). PON1 is an antioxidant enzyme found in the membranes of most cells and, as well, in the circulation bound to high-density lipoproteins (HDL). The original function attributed to PON1 is that of a lactonase since lipophilic lactones are part of its primary substrates. It is this catalytic capacity that enables PON1 to degrade lipid peroxides within the cell and in the lipoproteins in circulation. In addition, PON1 has an esterase activity and degrades organophosphate xenobiotics such as paraoxon, phenylacetate and nerve agents [11]. A recent meta-analysis showed decreased serum PON1 activity in patients with cancer, suggesting an impaired ability to combat oxidative stress, with potential implications in cell proliferation, promotion of genetic instability, and alterations in cellular sensitivity to chemotherapy [12]. We have shown previously that RT was associated to an increase in serum PON1 levels in cancer patients [13, 14].

The present article presents a pilot study on the feasibility of determinations of the circulating levels of PON1 and compounds related to energy metabolism as biomarkers for the evaluation of patients with RC, and the effects produced by NRCT.

## Materials and methods

### Participants

This was a longitudinal, prospective study of 32 patients with RC (men, n = 22; women, n = 10) admitted to our Hospital between March 2014 and June 2018. Tumours were classified according to the TNM staging system [15]. They were diagnosed with locally advanced RC (cancer stage II or III, and T3/4 and N0/1/2, according to the clinical TNM system). All patients accepted a clinical visit one month after the NRCT, but only a small subgroup of 14 patients agreed to have a new blood sample drawn. RT was administered five days per week, for 4–6 weeks, and the total radiation final dose was 50.4 Gy (1.8 Gy/day) in the pelvis, with a boost in the tumour area of 5.4 Gy. All participants received capecitabine concomitant chemotherapy together with the RT sessions. The Dworak system was used to estimate the tumour regression grade (TRG) after NRCT [16]. Surgery was carried out between 6 and 8 weeks after the end of NRCT. Blood samples were obtained from each patient before NRCT and one month after NRCT and before surgery. Serum and EDTA-plasma aliquots were stored at -80°C until batched biochemical determinations. For comparisons, biobanked samples (*n* = 48) were used from healthy, age-matched volunteers without liver alterations, whose details were previously described [17]. Procedures were approved by the Ethics Committee of *Hospital Universitari de Sant Joan* (Project 14/2014). All patients gave their written informed consent, provided that the anonymity of the data was guaranteed.

### Biochemical analyses

Serum PON1 activity was measured as the rate of hydrolysis of phenylacetate at 280 nm, in a 9 mM Tris-HCl buffer, pH 8.0, supplemented with 0.9 mM CaCl2, as previously reported [18]. Serum PON1 concentration was determined by in-house ELISA with rabbit polyclonal antibodies generated against the synthetic peptide CRNHQSSYQTRLNALREVQ, which is a sequence specific for mature PON1 [19]. The concentrations of metabolites involved in glycolysis, the citric acid cycle and amino acid metabolism were analysed by gas chromatography coupled with quadrupole time-of-flight mass spectrometry with an electron impact source (GC-EI-QTOF-MS), as described previously [20]. Analyses were performed with a 7890A gas chromatograph coupled with an electron impact source, to a 7200 quadrupole time-of-flight mass spectrometer equipped with a 7693 autosampler module and a J&W Scientific HP-5MS column (J&W Scientific HP-5MS column, 30 m × 0.25 mm, 0.25 μm, Agilent Technologies, Santa Clara, CA, USA). Calibration curves were obtained for each metabolite by plotting standard concentrations as a function of the peak area. Recovery of each metabolite ranged between 83% and 99%. Serum alanine aminotransferase (ALT) and aspartate aminotransferase (AST) activities, and CEA and CA19.9 were analysed by standard tests in a Roche Modular Analytics P800 system (Roche Diagnostics, Basel, Switzerland).

### Statistical analyses

For sample size calculation of comparison between patients and controls, we considered a mean difference ≥ 10 μg/L of PON1 concentration as an endpoint. Accepting an α risk of 0.05 and a β risk of 0.2 in a bilateral contrast, a minimum of 11 control subjects and 16 patients was required. Differences between any two independent groups were assessed using the Mann-Whitney U test. The Wilcoxon signed-rank test was used for comparisons of dependent variables. The Spearman correlation coefficient was used to evaluate the degree of association between variables. The combined effect of clinical and demographic characteristics on selected biochemical variables was assessed by ANOVA. All statistical calculations and graph representations were performed using the statistical package for social sciences (SPSS 22.0, Chicago, IL, USA) and GraphPad Prism 6.01 (GraphPad Software, San Diego, CA, USA). Significant differences were considered at a p-value < 0.05. Frequencies were expressed as medians and interquartile ranges for quantitative variables and as frequencies and percentages for qualitative variables. For metabolomics analysis, data were imported and analysed using MetaboAnalyst 3.0 (http://www.metaboanalyst.ca/) and the ‘R’ program version 4.0.2 (https://www.r-project.org/). Partial Least Squares Discriminant Analysis (PLSDA) was performed for multivariate analysis. The relative magnitude of observed variations was evaluated using the Variable Importance in Projection (VIP) score. Heatmaps and correlation matrices were included for a visual representation of individual metabolites and to summarise the predicted results. Spearman pairwise correlation matrices were analysed with the ggcorr package in R (https://www.rdocumentation.org/packages/GGally/versions/1.5.0/topics/ggcorr). Correlation networks were analysed to represent Spearman correlations between study groups, using the qgraph package of the Rstudio software. The threshold was determined at 0.05. To identify biologically significant patterns through metabolite concentration changes, we used a quantitative enrichment analysis (QEA) through MetaboAnalyst 4.0. This analysis was used to identify the metabolic patterns underlying significant changes in metabolite concentrations. QEA takes into account the counts and the calculated Q statistic for each metabolite for enrichment analysis [21]. The diagnostic accuracy of the measured biochemical variables was assessed by ROC curves [22].

## Results

### Clinical characteristics of RC patients

All raw clinical and biochemical data from this study are displayed in S1 Dataset, and the main clinical characteristics of the control group and patients with RC, pre- and post-NRCT, are shown in Table 1. Significant differences were observed in age, sex, dyslipidemia, arterial hypertension and body mass index (BMI) between patients with cancer and the control group. The BMI was higher in patients with RC and decreased after NRCT. The majority of tumours presented a medium location in the rectum (between 5 and 10 cm from the anal margin). Regarding clinical TNM classification (before receiving any oncological treatment), most of the patients were classified as T3, N1, M0, in the TNM system, stage III being predominant. Once the surgical piece was analysed, most patients had a pathological T3, N0, M0, and almost half of the surgical pieces showed a Dworak grade 3, indicating fibrosis with scattered tumour cells with/without acellular mucin. The toxic effects of treatment are shown in Table 2. Perianal dermatitis (Grade I) was the most prevalent early secondary effect. Neither of these patients showed late secondary effects. Most RC patients did not relapse once the cancer had been overcome and 81% of overall survival was observed over a four-year period as a median.

**Table 1:** Clinical characteristics of the control group and patients with Rectal Cancer (RC), pre- and post-Neoadjuvant Radiochemotherapy (NRCT).

Variables	Control group (n = 48)	RC pre-NRCT (n = 32)	RC post-NRCT (n = 32)
Age (years)	41.9 (10.0)	67.7 (9.5) ^c^	-
Women	29 (60.4)	10 (31.3) ^a^	-
Dyslipidemia	1 (2.1)	18 (56.3) ^c^	-
Arterial hypertension	2 (4.2)	18 (56.3) ^c^	-
Diabetes	4 (8.3)	5 (15.6)	-
Body mass index (kg/m^2^)	25.6 (4.4)	30.2 (4.2) ^c^	28.7 (4.3) ^b^^,^ ^d^
Smokers	13 (27.1)	7 (22)	-
Alcohol intake	11 (22.9)	9 (28)	-
Tumor localization			
High (10–15 cm)	-	11 (34)	-
Medium (5–10 cm)	-	15 (47)	-
Low (< 5 cm)	-	6 (19)	-
Clinical T (TNM system)			
T3	-	28 (88)	-
T4	-	4 (13)	-
Clinical N (TNM system)			
N0	-	7 (22)	-
N1	-	17 (53)	-
N2	-	8 (25)	-
Clinical M (TNM system)			
M0	-	32 (100)	-
Clinical Stage			
II	-	5 (16)	-
III	-	27 (84)	-
Pathological T (TNM system)			
T0	-	-	7 (22)
*In situ* T (Tis)	-	-	2 (6)
T1	-	-	1 (3)
T2	-	-	9 (28)
T3	-	-	11 (34)
T4	-	-	2 (6)
Pathological N (TNM system)			
N0	-	-	26 (81)
N1	-	-	5 (16)
N2	-	-	1 (3)
Pathological M (TNM system)			
M0	-	-	32 (100)
Dworak TRG			
1	-	-	4 (13)
2	-	-	7 (22)
3	-	-	16 (50.0)
4	-	-	5 (16)
Relapse			
None	-	-	24 (75)
Metastasis	-	-	5 (16)
Other neoplasms	-	-	3 (9)
Overall survival*	-	-	26 (81)
Disease free survival*	-	-	22 (69)
Follow-up (months)	-	-	48.0 (26.2–63.0)

Results are shown as frequencies and percentages (in parenthesis) except of age, BMI and survival, which are shown as means and standard deviations.

^a^ p < 0.05,

^b^ p < 0.01,

^c^ p < 0.001 with respect to the control group;

^d^ p < 0.05 with respect to RC pre-NRCT. TNM: tumor, node, metastasis staging system; TRG: tumor regression grade.

* Survival was estimated between 2 and 5 years after the clinical diagnosis, with a mean of 4 years.

**Table 2:** Early toxicity effects of neoadjuvant radiochemotherapy.

Toxicity effect	Number of patients
**Diarrhea**	
Grade I	2 (6)
Grade II	1 (3)
**Perianal dermatitis**	
Grade I	19 (59)
Grade II	8 (25)
Cystitis	
Grade I	5 (16)
Grade III	1 (3)
**Asthenia**	
Grade I	1 (3)

Results are shown as frequencies and percentages (in parenthesis).

### Alterations in PON1-related variables and other biochemical parameters

We observed significantly lower serum PON1 concentrations and activities in patients with RC pre-NRCT compared to the control group. Treatment was associated with an increase in PON1 concentrations. We did not find any significant differences in aminotransferase levels between patients and the control group, either before or after the therapy. Carcinoembryonic antigen (CEA) concentrations decreased after NRCT, but we did not find any significant differences in carbohydrate antigen 19.9 (CA 19.9) (Table 3). Differences in serum PON1 concentrations between RC pre-NRCT patients and the control group were maintained when adjusted for differences in clinical and demographic characteristics (Table 4).

**Table 3:** Selected biochemical variables in the control group and Rectal Cancer (RC) patients, pre- and post- Neoadjuvant Radiochemotherapy (NRCT).

	Control group (n = 48)	RC before NRCT (n = 32)	RC after NRCT (n = 14)
**PON1 related-variables**			
PON1 concentration (mg/L)	100.9 (88.4–132.8)	34.6 (24.3–57.8) ^c^	62.5 (28.2–97.1) ^b^^,^ ^d^
PON1 activity (U/L)	104.8 (89.3–126.0)	97.0 (80.0–104.5) ^a^	89.8 (78.0–98.6) ^a^
**Aminotransferases**			
ALT (U/L)	18.2 (14.4–28.8)	16.6 (13.0–19.0)	18.0 (12.5–21.5)
AST (U/L)	20.0 (17.7–24.1)	18.0 (15.0–19.0)	18.0 (15.0–21.0)
**Tumor markers**			
CEA (ng/mL)	-	4.4 (2.5–10.2)	2.6 (1.8–5.4) ^d^
CA 19.9 (U/mL)	-	11.8 (7.1–29.0)	10.2 (7.2–19.8)

Results are shown as medians (interquartile ranges).

^a^p < 0.05,

^b^p < 0.01.

^c^p < 0.001 with respect to the control group;

^d^p < 0.05 with respect to RC pre-NRCT.

ALT: alanine aminotransferase; AST: aspartate aminotransferase; CEA: carcinoembryonic antigen; CA 19.9: carbohydrate antigen 19.9; PON1: paraoxonase-1.

**Table 4:** Multiple regression analysis of the combined effect of clinical and demographic characteristics on serum paraoxonase-1 concentration.

	B	SE	t	p-value
Constant	120.512	35.989	3.349	0.001
Age	-0.118	0.530	-0.223	0.824
Sex	3.480	10.024	0.347	0.730
Dyslipidemia	6.895	17.368	0.397	0.693
Arterial hypertension	-6.783	14.788	-0.459	0.648
Diabetes	11.538	14.654	0.787	0.434
Body mass index (kg/m^2^)	-0.225	1.220	-0.184	0.854
Diagnosis (control or patient)	-65.510	17.678	-3.706	<0.001

Dependent variable, paraoxonase-1 (mg/L). Model summary: *r* = 0.697; p < 0.001.

### Variations in energy balance-associated metabolites

**Fig 1:**
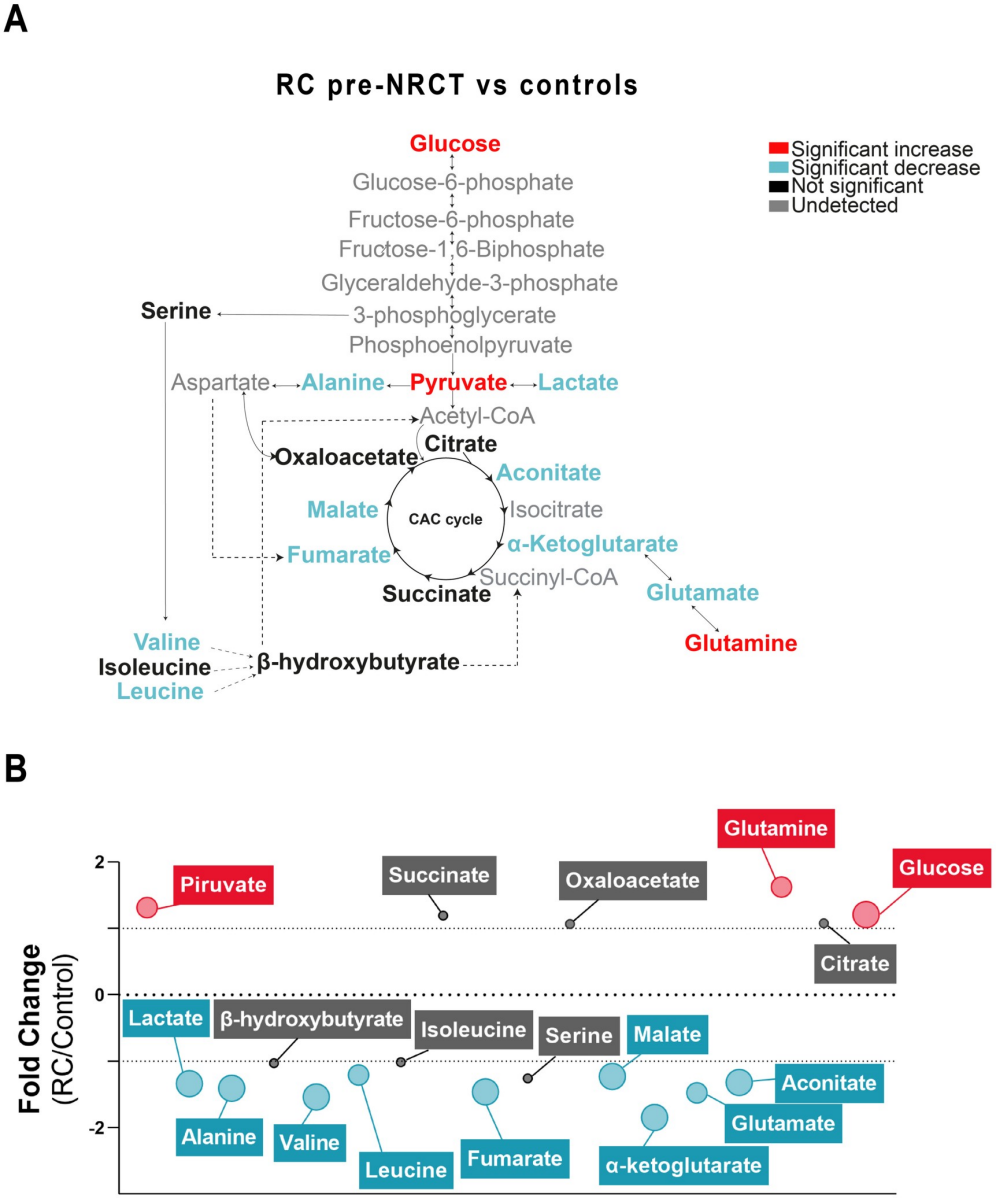
Plasma metabolic alterations in patients with Rectal Cancer (RC). (A) Schematized view of differences in metabolites related to the glycolytic pathway, citric acid cycle, and amino acid pathways between control and RC patients. Colours indicate statistical comparisons as shown in the legend. (B) Average fold changes of metabolite concentrations expressed as bubble plots. The bubble sizes estimate the magnitude of change. Colours represent a significant statistical increase (red), decrease (blue) or not significant (grey). NRCT: Neoadjuvant radiochemotherapy.

**Fig 2:**
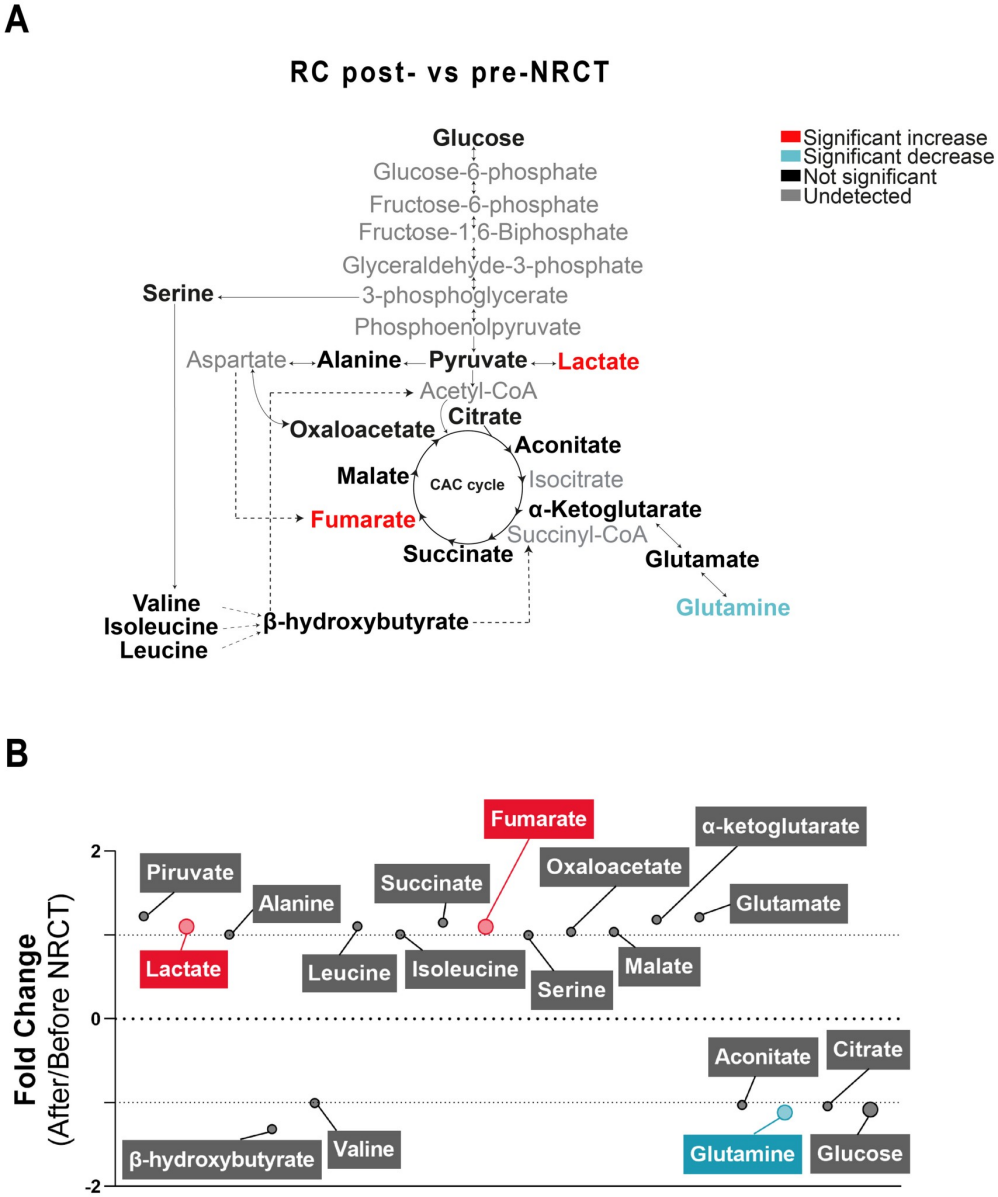
Plasma metabolic alterations in patients with Rectal Cancer (RC) after Neoadjuvant Radiochemotherapy (NRCT). (A) Schematised view of differences in metabolites related to the glycolytic pathway, citric acid cycle, and amino acid pathways between control and RC patients. Colours indicate statistical comparisons as shown in the legend. (B) Average fold changes of metabolite concentrations expressed as bubble plots. The bubble sizes estimate the magnitude of change. Colours represent a significant statistical increase (red), decrease (blue) or not significant (grey).

Changes in the concentrations of the analysed metabolites in RC patients pre-NRCT compared with the control group are shown in Fig 1. We found significantly higher concentrations of plasma glucose, pyruvate and glutamine in RC patients. In addition, lactate, alanine, valine, leucine, fumarate, malate, α-ketoglutarate, glutamate and aconitate concentrations were significantly lower (Fig 1A). The mean fold change of metabolite concentrations is also represented on a bubble plot. α-ketoglutarate and glutamine metabolites showed the biggest differences with mean fold changes of -1.84 and 1.62, respectively (Fig 1B). When comparing metabolic alterations post-NRCT *vs*. pre-NRCT, we found significant increases in lactate (1.1 average fold change) and fumarate (1.1 average fold change) and a decrease in glutamine (-1.1 average fold change) (Fig 2).

**Fig 3:**
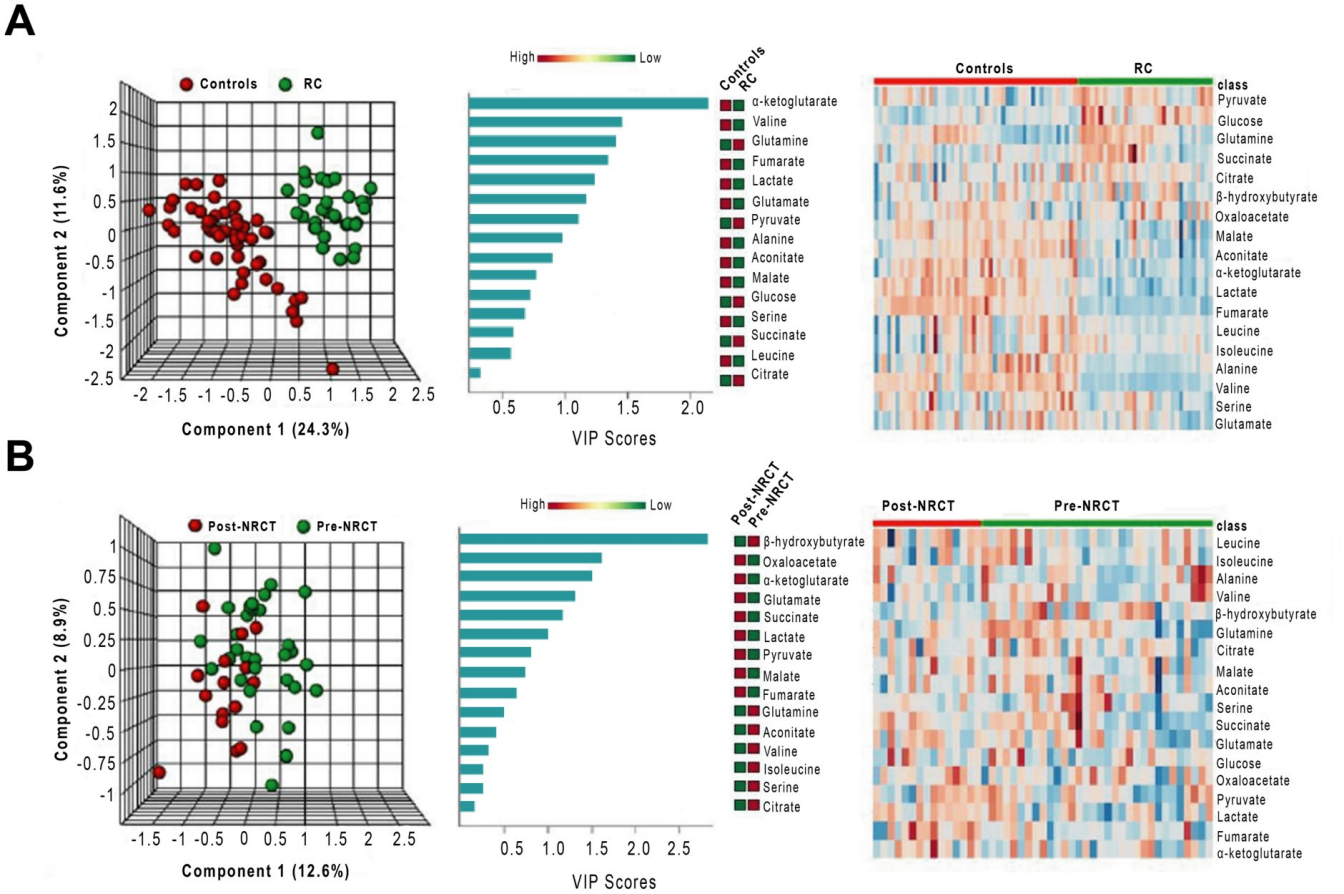
Multivariate analysis of plasma metabolites patients with Rectal Cancer (RC) and the control group pre- and post-Neoadjuvant Radiochemotherapy (NRCT). From left to right, partial least square discriminant analysis (PLSDA), variable importance in projection (VIP) scores of the PLSDA and heatmap analyses comparing RC patients and control group (A) and RC patients pre- and post-NRCT (B).

The three-dimensional representation of PLSDA showed that the combination of the analysed metabolites allowed a complete differentiation between control and RC pre-NRCT groups. Conversely, we found a considerable degree of overlapping when comparing patients pre- and post- treatment. VIP score indicated that the most relevant metabolite for distinguishing RC patients and the control group was α-ketoglutarate. Heatmaps showed a clear difference between the metabolite levels between patients and controls, but these differences were not so evident when comparing patients pre- and post-NRCT (Fig 3). Differences in plasma α-ketoglutarate concentrations between RC pre-NRCT patients and the control group were maintained when adjusted for differences in clinical and demographic characteristics (Table 5).

**Table 5:** Multiple regression analysis of the combined effect of clinical and demographic characteristics on plasma α-ketoglutarate concentration.

	B	SE	t	p-value
Constant	10.080	8.909	1.131	0.262
Age	0.180	0.132	1.362	0.178
Sex	-5.957	2.501	-2.382	0.020
Dyslipidemia	1.445	4.337	0.333	0.740
Arterial hypertension	-2.934	3.695	-0.794	0.430
Diabetes	-1.309	3.661	-0.358	0.722
Body mass index (kg/m2)	0.415	0.303	1.370	0.176
Diagnosis (control or patient)	-19.392	4.417	-4.390	< 0.001

Dependent variable, α-ketoglutarate (μM). Model summary: *r* = 0.647; p < 0.001.

### Relationships between PON1-related variables and energy balance-associated metabolites

**Fig 4:**
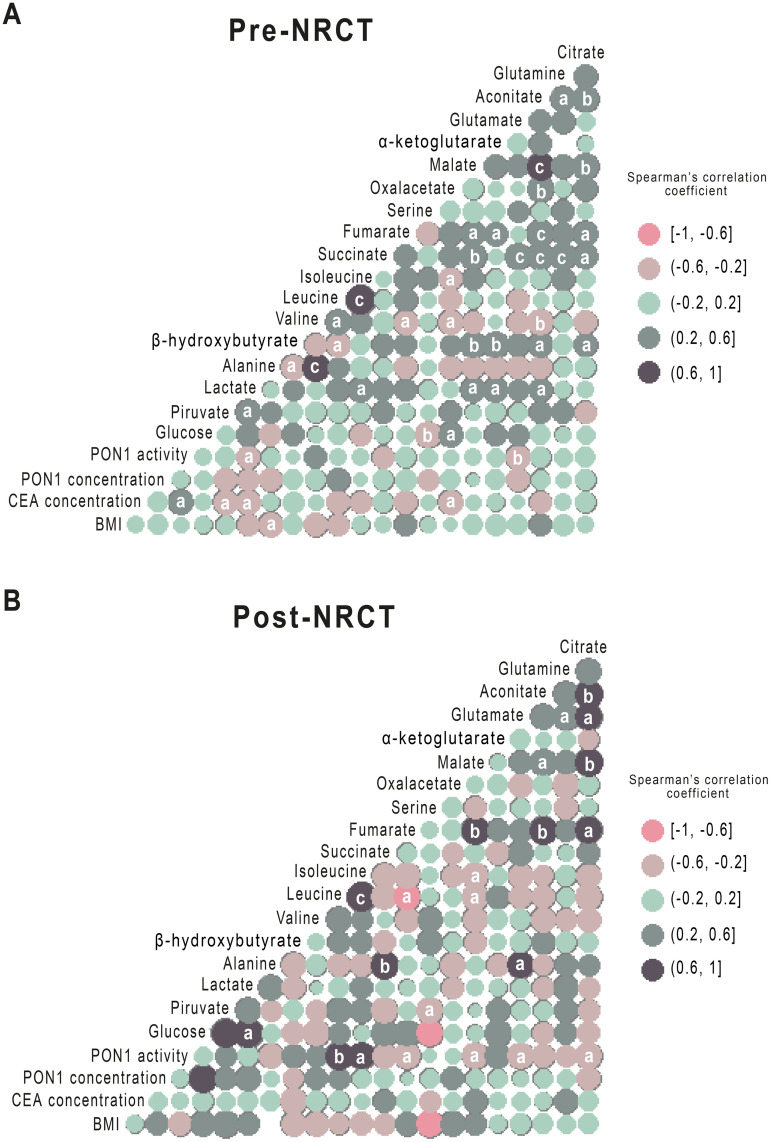
Spearman pairwise correlation matrices of metabolites and Paraoxonase-1 (PON1) activity and concentration pre- and post- Neoadjuvant Radiochemotherapy (NRCT). The magnitude and direction of the correlation of metabolites concentration pre- (A) and post- (B) treatment are shown by the circle size (larger is stronger) and colours (positive correlation in dark blue and negative correlation in pink), respectively. a = p<0.05; b = p<0.01; c = p<0.001.

Spearman’s correlations between the different metabolites pre- and post-NRCT are represented as correlation matrices (Fig 4). There were multiple significant correlations between the different metabolites both pre-NRCT and post-NRCT. Serum PON1 activity showed inverse relationships with glutamate and lactate pre-NRCT. In addition, direct correlations post-NRCT were found with leucine and isoleucine, and inverse correlations with glutamate, fumarate, citrate and malate.

#### PON1 and energy metabolism are linked with clinical characteristics of the RC patients

**Fig 5:**
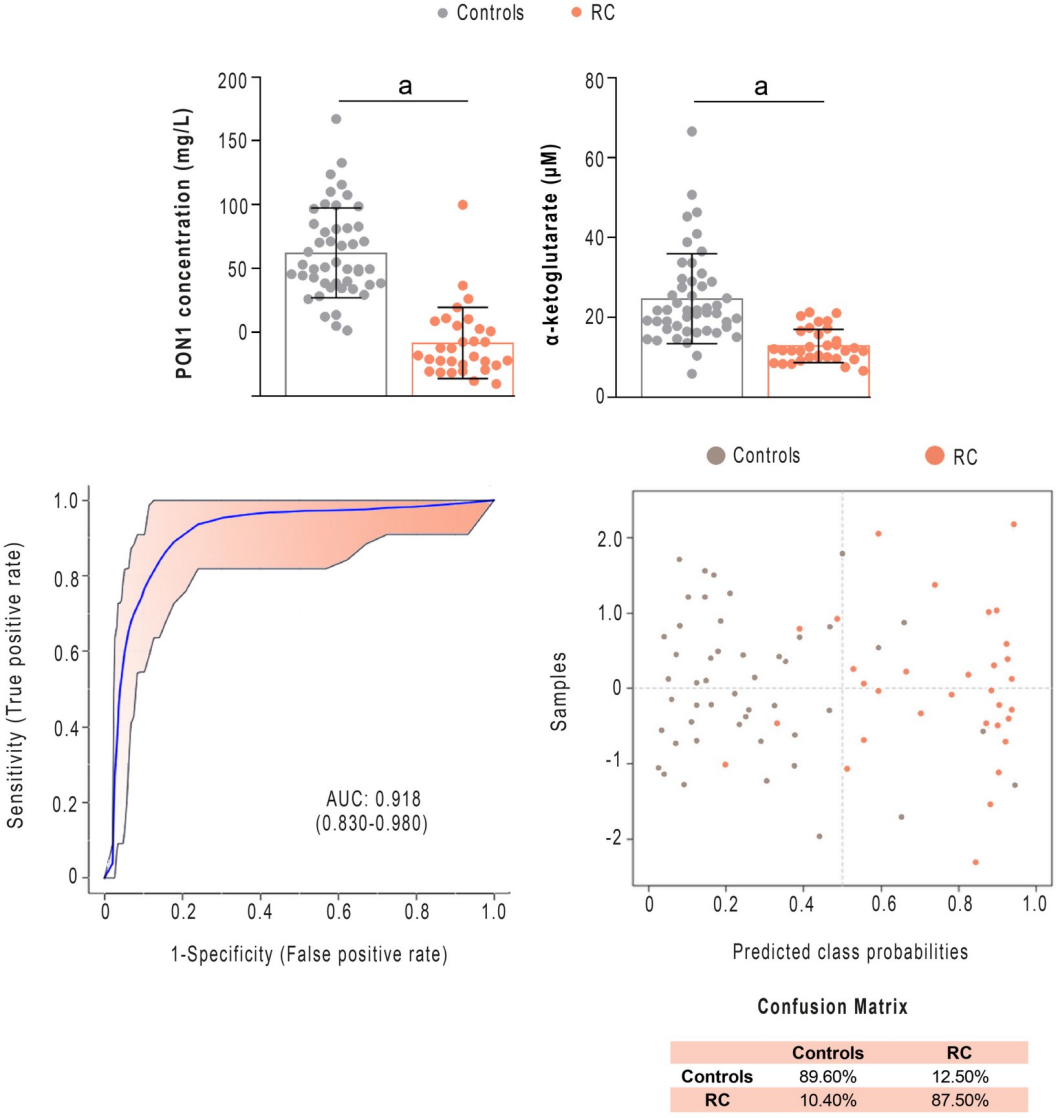
Serum Paraoxonase-1 (PON1) and α-ketoglutarate concentrations discriminate between patients with Rectal Cancer (RC) and the control group. PON1 and α-ketoglutarate concentrations were significantly lower in patients than in healthy subjects. Receiver operating characteristics curve-based model evaluation and confusion matrix showed that the combination of these parameters was able to efficiently distinguish both groups. AUC: Area under the curve. a = p<0.001.

The statistical analyses specified in the previous sections identified PON1 and α-ketoglutarate concentrations as the parameters that best discriminated between RC and the control group. Receiver operating characteristics (ROC) analysis of the combination of both parameters showed a very high diagnostic accuracy, with an area under the curve (AUC) greater than 0.918 and a correct classification of the patients in more than 87% of the cases, according to the confusion matrix (Fig 5) In addition, we observed significant differences (p = 0.003) in α-ketoglutarate concentrations between patients with clinical stage II [19.0 μM (16.4–20.7)] and stage III [11.5 (9.4–12.9)] but not in PON1 concentration [stage II: 27.7 mg/L (10.6–50.5)] and stage III: [37.3 (24.8–60.4)], p = 0.243).

**Fig 6:**
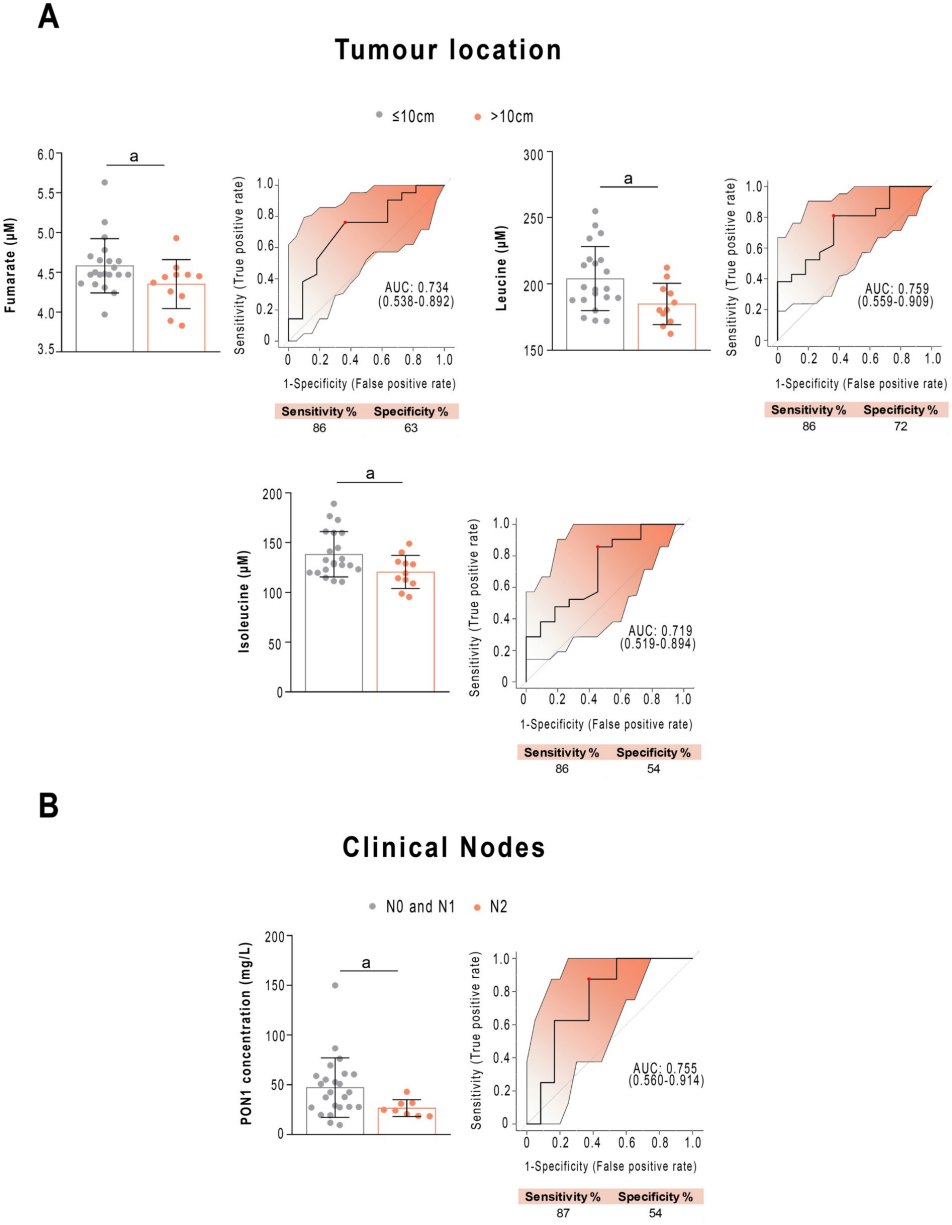
Selected metabolites and Paraoxonase-1 (PON1) concentrations are associated with clinical variables before treatment. (A) Receiver operating characteristics curve-based model evaluation showing that the combination of fumarate, leucine and isoleucine was able to efficiently distinguish tumour location near to (<5cm) or far from (>10cm) the anal margin in patients with rectal cancer. Confusion matrix showing the performance summary of the predicted results. (B) Receiver operating characteristics curve-based model evaluation showed that PON1 concentration was able to discriminate between patients with Nodes 0+1 *vs*. Node 2. Sensitivity and specificity are shown as percentages. AUC: Area under the curve. a = p<0.05; b = p<0.01.

Next, we wanted to find out which analytical parameters were related to the main clinical characteristics of the patients. Regarding the tumour location, the pre-NRCT concentrations of fumarate, leucine and isoleucine were lower in patients if the tumour was located far from the anal margin. ROC analysis showed AUCs higher than 0.70, with a wide confidence interval, sensitivity of 86% and specificity ranging from 54 to 72% (Fig 6A). Regarding clinical N, patients diagnosed with N2 had a lower PON1 concentration pre-NRCT than those diagnosed with N0 or N1. ROC analyses revealed that PON1 concentration allowed identification of the patients with two affected positive nodes (including the presence of tumour cells in 4 or more regional lymph nodes), with an AUC value of 0.755 (sensitivity = 87%, specificity = 54%, Fig 6B).

**Fig 7:**
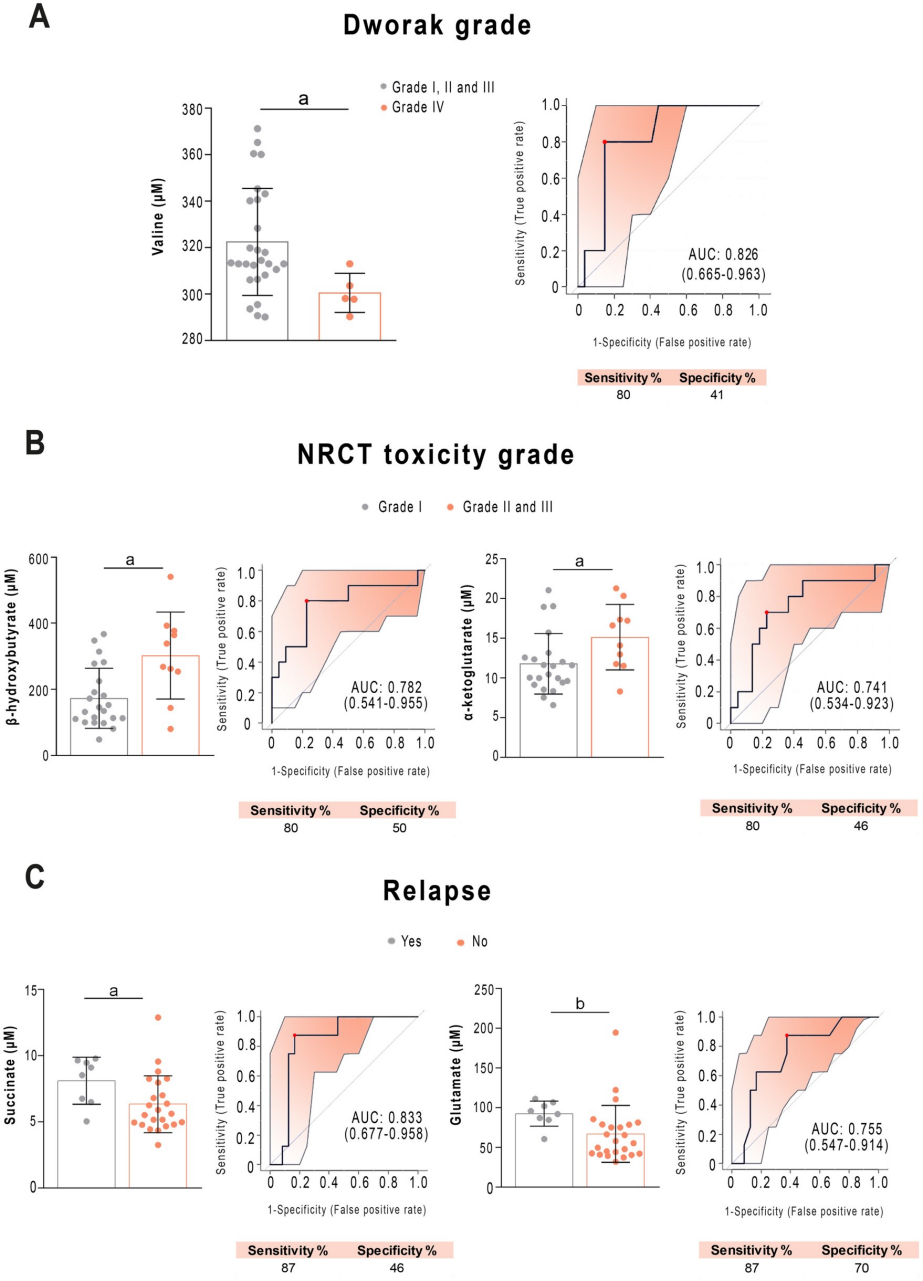
Selected metabolites predicting the prognosis of patients with rectal cancer before treatment. Receiver operating characteristics curve-based model evaluation showing that valine was related to Dworak grade (A), β-hydroxybutyrate, α-ketoglutarate were related to neoadjuvant radiochemotherapy (NRCT) toxicity grade (B), add succinate and glutamate were related to tumour relapse (C). Sensitivity and specificity are shown as percentages. AUC: Area under the curve. a = p < 0.05; b = p < 0.01.

Of great clinical relevance is whether any of the analysed parameters allow the prediction in advance of the patients who would present a pathological complete response (pCR) to treatment. We found that patients who responded efficiently had lower plasma pre-NRCT valine concentrations than patients who responded partially or not at all. ROC analysis showed an AUC of 0.826 (sensitivity = 80%, specificity = 50%) (Fig 7A). In contrast, we din not observe any significant relationship between PON1 concentration and the pathological response or relapse. When investigating relationships of toxicity to NRCT, we found that the pre-NRCT concentrations of β-hydroxybutyrate and α-ketoglutarate were higher in patients who presented grade II and III acute toxicity compared to grade I (Fig 7B), showing AUC values higher than 0.70 (sensitivity = 80%, specificity = 0.50). Finally, when investigating relationships to tumour relapse, we observed that patients who relapsed during the follow-up period had lower concentrations of succinate and glutamate (Fig 7C). ROC curves showed an AUC = 0.833 (sensitivity = 87%, specificity = 48%) for succinate, and AUC = 0.755 (sensitivity = 87%, specificity = 70% for glutamate).

**Fig 8:**
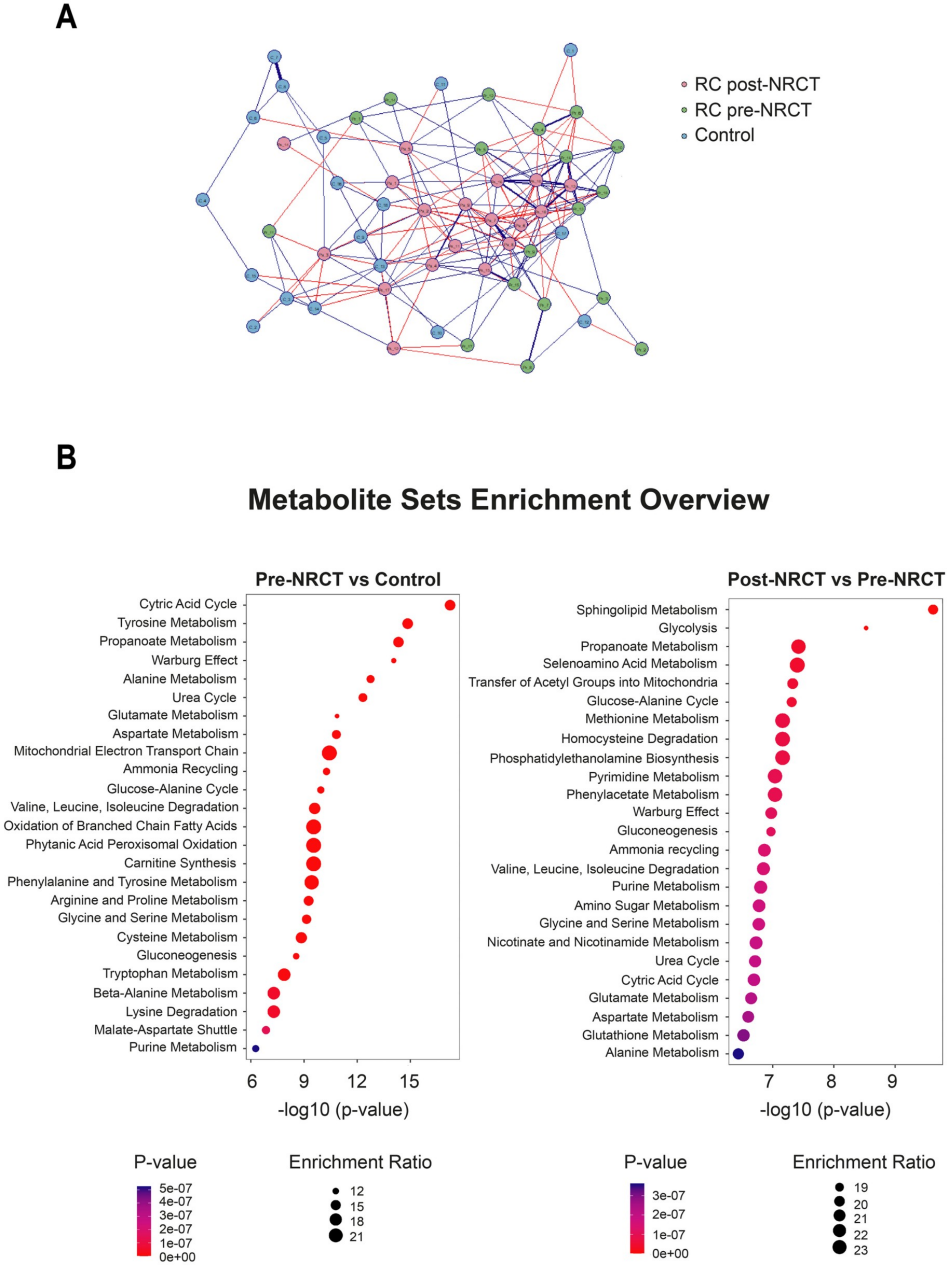
Correlation-based network of metabolite concentrations from control subjects and patients with Rectal Cancer (RC) pre- and post-neoadjuvant radiochemotherapy. The Spearman correlation was applied to estimate correlation coefficients, and threshold tests were used to identify significant correlations with a 0.05 False Discovery Rate cutoff. Metabolites are shown as circle nodes with three different colors (pink, green and blue), depending on the group. Positive and negative correlations are illustrated as blue or red edges, respectively. Strongest correlations are visualized with thick lines (A). Metabolite Sets Enrichment analysis from metabolite concentrations. Dot plots were used to represent the Quantitative Enrichment Analysis. The size and color of the dots show the count of enriched metabolites in the pathway and the importance of the pathway enrichment, respectively (B).

Fig 8 represents a correlation network of the grouping of metabolites from each study group. We identified some stronger commonalities and positive connections in the post-NRCT group compared to the control and pre-NRCT groups (Fig 8A). We drew dot plots to show the precise influence of each individual metabolite to the total score for the pool of metabolites (Fig 8B). When comparing the control group with the RC patients pre-NRCT, we observed that the most relevant pathways in metabolic alterations were the citric acid cycle and tyrosine metabolism. However, when we compared the effects of NRCT, we observed that sphingolipid metabolism and glycolysis were the most prominent pathways.

## Discussion

In recent years, various studies have been conducted to identify RC biomarkers in biological fluids using metabolomics methods [23–25]. However, to the best of our knowledge, the present study is the first to investigate alterations in energy metabolism and its relationship with PON1. Our results show that determining circulating levels of both PON1 and α-ketoglutarate can differentiate patients from healthy subjects with a great level of accuracy.

PON1 is one of a family of three enzymes, called PON1, PON2, and PON3, which degrade lipid peroxides in circulating lipoproteins and in the cytoplasmic and intracellular organelle membranes of cells [11]. These enzymes are linked to mitochondria-associated membranes, they modulate mitochondrial metabolism, and they prevent apoptosis. Their overexpression protects mitochondria from endoplasmic reticulum stress and subsequent mitochondrial dysfunction; highlighting that the anti-inflammatory effects of paraoxonases may be mediated, at least in part, by their protective role in mitochondria and associated organelle function [26–28]. In the present study, we found a decrease in PON1 concentration and activity in RC patients pre-NRCT compared with control subjects. These results agree with previous studies that reported low serum PON1 activity in patients with colorectal cancer [29–31]. Serum PON1 activity correlated to some energy balance-related metabolites, which could be a reflection of the peripheral circulation of the protective effect of this enzyme on the mitochondria or that alterations in mitochondrial metabolism affect the activity of the enzyme both within the cell and in circulation, as it has been described in other chronic diseases [32]. NRCT was linked to an increase in the concentration of this enzyme, but not in its activity. This disparity is not surprising since, to hydrolyze lipid peroxides, PON1 must covalently bind to lipid molecules [33]. The result is that the PON1 molecule is inactivated [34]. Therefore, an increase in oxidative stress, such as that produced by NRCT, would be associated with a partial decrease in PON1 activity, which does not correlate with the increase in its concentration. We observed significant correlations between the levels of PON1 activity and some energy balance-related metabolites, a logical observation considering the relationship of this enzyme with protection against oxidative stress and mitochondrial function. An association that was maintained both before and after NRCT was the negative relationship between PON1 activity and glutamate concentration. Some studies suggest that glutamate is pro-oxidant and pro-inflammatory [35], so it is logical that an increase in circulating glutamate levels is associated with a decrease in PON1 activity through the mechanisms discussed above. One of the most important alterations is a marked decrease in the pre-NRCT plasma concentration of α-ketoglutarate. Changes in the levels of α-ketoglutarate have been observed in several classes of cancer [36–39]. This compound is a product of glutaminolysis, a biochemical pathway that stimulates tumour development, including protein and nucleic acid syntheses, epigenetic changes, metabolite exchange between the mitochondria and the cytosol, and the stimulation of antioxidant defence mechanisms [40, 41]. We found a significant decrease in plasma α-ketoglutarate concentrations in RC patients pre-NRCT, which probably reflects an increase in its intracellular demand.

The AUC of the ROC curves of the combined measurement of pre-NRCT PON1 and α-ketoglutarate concentrations in the distinction between patients with RC and healthy subjects was 0.918, which compared favourably with other biomarkers studied in recent years. For example, an AUC of 0.81 has been reported for a panel of 5 methylated microRNAs [42], while for the determination of small proline-rich protein 2A it was 0.851 [43], for urinary 8-OH-guanosine it was 0.755 [44], and for free fatty acids it was 0.61 [45]. From this perspective, our results suggest that the measurement of PON1 and α-ketoglutarate concentrations pre-NRCT could be a useful tool for the early diagnosis of RC.

NRCT before surgery is the commonly used treatment for RC due to its proven benefit in reducing the rate of local recurrence, but the response is highly variable, with approximately 20% of patients presenting with a pCR, and up to 40% showing no regression or even tumour progression [46]. The ability to predict the response to NRCT could crucially affect any decision made about the treatment of patients. It would be interesting to know in advance which patients might not respond well to NRCT, because they could proceed directly to surgery, avoiding treatment delay. On the other hand, patients predicted to have a pCR would be the better choice for NRCT and could even be considered for non-operative management, thus avoiding the significant risk of death and morbidity associated with RC surgery [47]. Clinical factors, including tumour size, clinical T and N stages, distance of the tumour from the anal verge, and the interval from NRCT to surgery, all effect the response to treatment [48–52]. Many efforts have been made to identify clinical or molecular predictors of pCR following NRCT in rectal cancer patients and this is an active and promising line of research [46, 53, 54], but there are not many articles investigating the utility of metabolomics methods. Jia et al. [55] have recently reported that a panel of 57 metabolites is able to discriminate between sensitive and resistant patients to NRCT with an AUC of the ROC curve of 0.81 using random forest analysis. The present study shows that patients with a pCR (Dworak Grade IV) have lower pre-NRCT plasma concentrations of the branched-chain amino acid valine than those presenting a partial response (Dworak Grades II and III), and that the measurement of this amino acid predicted pCR with 80% sensitivity and 41% specificity, and an AUC of 0.826. The reasons for this observation cannot be deduced from the present study, but it can be hypothesized that the lower values of circulating valine might be explained by a higher uptake by tumour cells due to a more active metabolism. The metabolism of most cancers relies on the use of glutamine catabolism for the replenishment of the mitochondrial citric acid carbon pool, which is required for the synthesis of lipids, proteins, and nucleic acids. Branched chain amino acids provide a major source for the biosynthesis of glutamine and glutamate. The increased demand on glutamine would explain the reduced plasma valine concentrations in actively replicating tumour cells. We suggest that the most metabolically active tumours would be those that respond best to NRCT and that this can be predicted before treatment by measuring the plasma valine concentration. Indeed, this is what is described in breast cancer, where patients with a triple-negative phenotype, with more metabolically active and more aggressive tumours, are the ones who respond best to chemotherapy treatment [56]. Interestingly, in our study, direct correlations between branched-chain amino acid levels and PON1 activity were observed, suggesting that patients with more metabolically active tumours have lower enzyme activities, perhaps due to increased oxidative stress.

Our results in relation to metabolic alterations and tumour location, the number of nodes and toxicity, are modest and the observed alterations seem of little relevance, given the little capacity to discriminate between the different conditions. Perhaps the most interesting result is that patients with tumour relapse had higher concentrations of plasma succinate. This can reflect a decrease in succinate dehydrogenase activity, an enzyme that is an integral component of the mitochondrial complex II and that catalyses the oxidation of succinate to fumarate with the reduction of ubiquinone to ubiquinol. Succinate dehydrogenase is the bridge enzyme between the citric acid cycle and the electron transport chain. Previous studies have reported that its activity is reduced in colorectal cell cultures and that its restoration inhibits the growth of cancer cells both *in vitro* and *in vivo* [57]. The loss of enzymatic activity affects the electron flow to ubiquinone, with an increase in oxidative stress and pro-oncogenic DNA damage [58]. On the other hand, accumulation of succinate transforms it into an oncometabolite that competitively inhibits α-ketoglutarate-dependent dioxygenase due to the structural similarity between succinate and α-ketoglutarate. The consequence is epigenetic changes giving rise to a hypermethylated DNA profile [59]. In addition, there is a relationship between the location of the tumour and the efficacy of treatment, with distal tumours responding worse [60]. In our study, the finding that these patients have lower circulating concentrations of fumarate, leucine, and isoleucine could indicate a greater use of these molecules by more metabolically active tumours.

The results obtained with the Quantitative Enrichment Analysis raise more questions than answers. However, some conclusions can be drawn. We have identified the citric acid cycle and tyrosine metabolism as the most relevant pathways in the distinction between RC patients and healthy subjects, and sphingolipid metabolism and glycolysis as the pathways that are most prominently altered by NRCT. These results are in agreement with previous studies. For example, the tyrosine kinase pathway has been associated with angiogenic signaling and systemic dissemination in patients with RC [61], while determinations of plasma concentrations of tyrosine, phenylalanine, and tryptophan have been proposed as biomarkers of gastroesophageal cancer [62]. Likewise, strong evidence suggests that sphingolipids regulate tumour cell apoptosis and may condition the response to radiotherapy or chemotherapy in various types of cancer [63, 64]. On the other hand, the importance of glycolysis and the citric acid cycle in neoplasms and its relationship with the aggressiveness of the tumour and its response to treatment is well known, since these pathways are the main sources of energy and substrates for the synthesis of proteins, lipids and nucleic acids of tumour cells [65].

A limitation of the present study is that being a pilot study, the number of patients studied is small and several observed findings may be due to chance. Multicenter studies with a larger number of patients are needed to reach definitive conclusions in this regard.

## Conclusion

The results of the present study illustrate the usefulness of investigating alterations in oxidative stress and metabolism in RC. Due to the small number of patients studied, our results must be considered preliminary, but they suggest that the determination of circulating levels of PON1 and α-ketoglutarate might be a valuable tool for the early diagnosis of RC, while the determination of valine and succinate might effectively predict pCR and the appearance of relapse.

## Supporting information

S1 Dataset Raw clinical and analytical data. (XLS)
